# Sport injury prevention in-school and out-of-school? A qualitative investigation of the trans-contextual model

**DOI:** 10.1371/journal.pone.0222015

**Published:** 2019-09-06

**Authors:** Alfred S. Y. Lee, Martyn Standage, Martin S. Hagger, Derwin K. C. Chan

**Affiliations:** 1 School of Public Health, The University of Hong Kong, Hong Kong Special Administrative Region, China; 2 Department for Health, University of Bath, Bath, United Kingdom; 3 Department of Psychological Sciences, University of California, Merced, California, United States of America; 4 Faculty of Education and Human Development, The Education University of Hong Kong, Hong Kong Special Administrative Region, China; 5 School of Psychology, Curtin University, Perth, Australia; University of Rome, ITALY

## Abstract

**Objective:**

To investigate junior secondary school students’ experiences and perspectives of in-school and out-of-school sport-safety, with a particular focus on the meaning and content that they applied to the motivational and social cognitive factors of sport injury prevention.

**Design:**

Focus-group interview.

**Method:**

Participants were 128 junior secondary school students (Form 1 to Form 3) aged between 12 and 16 years from two secondary schools. We organised focus-group interviews by class (group size = six to nine students). Seventeen groups completed semi-structured interviews regarding their experience, beliefs, and motives for injury prevention in-school and out-of-school. We analysed data by thematic content analysis using a typological approach.

**Results:**

Higher order themes (N = 7) including in-school and out-of-school motives and social cognitive factors and associated lower-order themes (N = 16), emerged from the analysis corresponding to constructs from trans-contextual model tenets.

**Conclusions:**

The current study is the first qualitative study to explore junior secondary school students’ experience and perspectives on sport injury prevention, using trans-contextual model as a framework for investigation. The findings contribute to a better understanding on their motivational and social cognitive factors in adopting sport injury prevention. The content of the theme behavior also indicated the inadequacy of students’ knowledge of effective sport injury prevention techniques, and underscored the importance of sport safety education.

## Introduction

Sport injury is one of the leading causes of injury in young people [1–3]. Not only may injury lead to temporary impairment of sport performance and absence from sport and school, but it could also result in prolonged pain, higher risk of re-injury, early retirement from competitive sport, and lower future commitment to physical activity for health [4–6]. Emery and Tyreman [1] reported that over 60% junior high school students (aged 12–15 years) suffered at least one sport injury in the past year. They also found that few injuries occurred during physical education (PE) classes. Most occurred in a game (39.3%) or recreational setting (26.9%), such as informal sport play in community parks. It therefore appears that sport injuries occur most often in out-of-school contexts.

Sport injury prevention includes static stretching, warm-up before and cool down after exercise, strength and conditioning [7], landing technique [8], and correct application of protective equipment (e.g., helmet) [9]. Sport injury prevention programs are provided for youth both in-school [10] and out-of-school [11]. Yet, the prevention of sport injury is a behavior that requires motivation and perseverance to maintain [12–14], particularly when students are unsupervised in out-of-school contexts (e.g., playing physically active games, leisure sport events). It is therefore important to understand why and how students learn sport safety in-school (PE lessons) and apply sport injury prevention in out-of-school contexts. In the current study, we employed a qualitative investigation guided by the trans-contextual model (TCM) [15–17] to explore and gain a rich understanding of the psychological processes underpinning students’ learning and application of sport safety principles. For the in-school context, we are referring to the PE lesson; out-of-school refers to both supervised and unsupervised physical activities.

The TCM integrates three important social psychological theories: including self-determination theory (SDT) [18, 19], the theory of planned behavior (TPB) [20], and the hierarchical model of intrinsic and extrinsic motivation (HMIEM) [21]. The pattern of motivation posited in TCM is classified generally by three forms of motivation and their sub-types defined by SDT [18, 19]. Autonomous form of motivation is an inherent drive to engage particular behaviors. Individuals are autonomously motivated when they are performing behaviors under intrinsic (e.g., acting for fun and pleasure), integrated (e.g., acting for behavior that is synthesis with own self) and identified (e.g., acting for achieving personally valued goal) motivation. In contrast, behaviors driven by externally-referenced reasons are known as controlled motivation which comprises introjected (e.g., acting to satisfy pride and ego, and avoid shame and guilt) and external (e.g., acting for compliance and to avoid punishment) regulation. Last but not least, amotivation refers to the absence of the motivation (e.g. acting for behaviors without any reason). The fundamental premise within the TCM is that the quality and quantity of motivation (i.e., autonomous, controlled motivation, and amotivation) based on tenets within SDT can be transferred from one context (e.g., taking PE lesson) to another related context (e.g., leisure-time physical activity), leading to changes in the social cognitive factors (i.e., attitude, subjective norm, perceived behavioral control (PBC), and intention from the TPB) that relate to intention, and actual behavioral participation. The proposition of TCM regarding the transferability of motivation is built on the assumption derived from the HMIEM [21]. The HMIEM proposes that forms of motivation from SDT operate at the three levels of generality (i.e., specific, contextual, and global) and are hierarchically related to each other. The motivational and behavioral patterns in one context are then expected to activate similar motivational patterns in allied behaviors in related contexts [22].

The original application of the TCM lies within transferring motivation between PE and leisure-time physical activity [15]. It was found that when students endorsed autonomous forms of motivation (i.e., identified regulation, and intrinsic motivation) rather than controlled forms of motivation (i.e., introjection and external regulation) in PE, they were more likely to be autonomously motivated toward leisure-time physical activity. Autonomous forms of motivation in leisure-time physical activity then predicted intention and self-reported physical activity via the mediation of the social cognitive factors. This pattern of results has been shown to be consistent across 12 countries, supporting the cross-cultural invariance of the original application of the TCM in PE and physical activity contexts [16, 17]. Therefore, promoting autonomous motivation of students in PE (e.g., PE teachers who support the psychological needs of students and their volitional engagement with PE activities; [16, 17] might be meaningful not only to the motivational pattern in an in-school context, but also to the motivational and social cognitive process associated with the behaviors in an out-of-school context.

Researchers have extended the potential of the TCM model to other behaviors, including rehabilitation [23], occupational injury prevention and rehabilitation [24], in-school and after-school learning [25], anti-doping in sport [26] and elite athletes’ sport injury prevention [27]. The trans-contextual process of motivation tested in these studies explains how motivation at work, school, or sport can be transferred into motivational, social cognitive and behavioral patterns of a related behavior in an allied context (e.g., rehabilitation for occupational injury, learning in out-of-school, sport injury prevention). In support of the tenets within TCM in the context of sport injury prevention, Chan and Hagger [27] found that elite athletes who possessed high autonomous motivation in sport tended to hold higher autonomous motivation for sport injury prevention. Autonomous motivation for sport injury prevention is a predictor of a wide range of behavioral outcomes of sport safety or injury prevention, such as adherence and commitment to injury prevention, prioritization and fatalism towards safety, and communication barrier and worry towards sport injury [27, 28]. Aligned with TCM predictions, the relationship between autonomous motivation for sport injury prevention and intention has been shown to be mediated by social cognitive variables [28, 29]. The TCM has been used to explain motivation and social cognitive process of human behaviors, including sport injury prevention, yet research has predominantly used quantitative methods to test the model. To date, extant work has not formally examined if the model is well-placed to explain students’ learning and application of sport safety in-school and out-of-school contexts. Somehow, it would not be comprehensive to understand students’ experience and perspectives in learning sport safety by using quantitative data only [30]. Hence, we proposed to adopt qualitative methodology to supplement existing research findings predominantly based on quantitative data.

In the present study, we employed qualitative methods to investigate junior secondary school students’ experiences and perspectives of in-school and out-of-school sport-safety, with a particular focus on the meaning and content that they applied to the motivational and social cognitive factors of sport injury prevention. The purpose of the present study was to explore the applicability and provide a holistic view of the TCM in secondary school students learning sport safety. Our study targeted junior secondary school students (Secondary 1 to Secondary 3, typically aged 12 to 16 years) because it is the beginning stage of secondary school education, a time in which sport safety is especially important for reducing the risk of sport injury in the later stages of PE [31, 32]. We conducted semi-structured focus group interviews with students to explore the content of the psychological variables in the TCM. We also examined number of codes identified respectively for autonomous motivation, controlled motivation, and amotivation. We were particularly interested to explore 1) the applicability of adopting TCM to explain secondary school students’ psychological factors underpinning sport safety; and 2) what are the realistic psychological processes of students learning sport injury prevention (e.g. particular reasons of adopting sport injury prevention, feelings or beliefs toward the prevention exercises) in-school and out-of-school. These analyses led to the first qualitative investigation of the TCM on sport safety in a junior secondary school setting. The results that obtained from the qualitative study would be useful to advance the understanding of TCM constructs in the context of sport safety for secondary school students, and the findings might inform the development of theory-driven interventions for sport injury prevention in school settings.

## Method

### Participants

Upon ethical approval from the first author’s institution [approval number = EA1604014], we conducted 17 focus-group interviews (6 to 9 participants per group) corresponding to a total number of 128 junior (Form 1 to Form 3 which are equivalent to 7^th^ to 9^th^ grade in US) secondary school students (69 males and 59 females; age = 12 to 16 years old; mean-age = 13.76, *SD* = 1.50) from two secondary schools in Hong Kong. Participants attended two mandatory PE lessons per teaching week. Most participants reported a history of sport injury (52.80%) such as a scrapes, sprained ankle, strained muscle, ligament rupture, or bone fractures. Some participants had experienced a sport injury in the last 6 months (18.75%). The variation in participants’ background in terms of age, gender, sport participation and injury experience enabled diverse perspectives of sport safety for enriching interview conversation [33]. The characteristics of each focus group are shown in Table 1.

**Table 1:** Focus group characteristics.

Groups	School	Form	Age	N	Gender
1	A	F1	Range = 12–13 (M_age_ = 12.50, *SD*_age = ._50)	28	Male = 13 Female = 15
2					
3					
4					
5	A	F2	Range = 13–15 (M_age_ = 13.69, *SD*_age = ._54)	28	Male = 14 Female = 14
6					
7					
8					
9	A	F3	Range = 14–15 (M_age_ = 14.60, *SD*_age = ._51)	35	Male = 20 Female = 15
10					
11					
12					
13	B	F2	Range = 13–16 (M_age_ = 13.94, *SD*_age =_ 1.89)	18	Male = 10 Female = 8
14					
15	B	F3	Range = 14–15 (M_age_ = 14.50, *SD*_age = ._94)	17	Male = 11 Female = 6
16					
17					

Note. The two local schools are marked as A and B to protect confidentiality and anonymity. M = mean; *SD* = standard deviation.

### Procedure

Secondary school students (Form 1 to Form 3) aged between 12 to 16 who attend regular PE lessons were invited to the study. Eligible students and their parents/guardians provide informed consent before the study. Students were asked to complete a short demographic questionnaire (e.g., age, gender, sport and sport injury experience) before joining the focus group interview. To foster a friendly environment in which students would freely interact with their peers, each focus group interview was formed by students within the same class, used the mother language of participants (i.e., Cantonese, the primary Chinese dialect in Hong Kong) as the medium of communication, and was moderated by one of the five Cantonese-speaking interviewers, including the first and second author, and three research assistants trained to follow the study protocol and moderate the interview according to the interview schedules. To enhance the quality and consistency of interview delivery, five interviewers ran 2 practice trials among themselves before the data collection.

At the beginning of the focus groups, interviewers raised questions about sports experience and motivation to play sports to establish rapport with the participants. Interviewers then provided a clear definition of sport injury (i.e., ‘any unintentional or intentional damage to the body resulting from participation in sport [34] and examples (e.g., abrasion, sprain, dislocation, or bone fracture), before leading the main topic of discussion to sport injury. Interviewers would then explore students’ sport safety knowledge by asking “What do you normally do to prevent sport injury in-school/out-of-school?”. The main part of the interview centered on questions about students’ motivation and social cognitive factors of sport injury prevention in-school and out-of-school. Examples of questions included “Why do you prevent sport injury in-school/out-of-school?”, “What are the pros and cons of doing sport injury prevention?” and “Under what circumstances, is sport injury prevention more difficult/ easy?”. The whole interview schedule is presented in S1 Appendix. The interviewers facilitated the discussion by (1) encouraging every group member to be active in contributing to, but not dominating, the interview, (2) asking for clarification and elaboration on certain points, (3) providing probing questions (e.g., “How do you feel”, “What do you think?”) to stimulate reflection of thoughts and feelings. At the end of the interview, participants were asked to discuss any additional issues that came to their mind about safety and injury prevention in sport. The focus group interviews lasted for 35 to 50 minutes with audio recordings transcribed verbatim.

### Data analysis

We adopted and followed Keegan and colleagues’ key analytical procedures [35, 36] in our qualitative data analysis, including (1) transcribing of interview content into 65 pages of single-lined text with 11 font size; (2) reading the transcript and listening to the interview recordings multiple times to increase familiarity; (3) conducting a thematic content analysis with typological approach [37] using ThematiCoder version 1.0 [38], and quotes could be coded into multiple themes; (4) checking consistency of all the coding with agreement of 96% between two coders; (5) paraphrasing and restating participants’ responses to ensure correct understanding and precise transcription of the data; (6) adopting a ‘critical friend’ approach to allow the two coders to critically review and challenge each other’s coding, categorization, organization, reflection, and interpretation of qualitative findings [39, 40], and (7) conducting a peer debriefing session among the research team members about the analysis. The essence of the thematic content analysis in this study was to systematically organize the lower-order themes that emerged inductively into higher order themes based on motivational and social cognitive factors of the TCM, so deductive data analysis would progressively take place until theoretical saturation was reached. Chi-square tests of independence examine if the frequency (i.e., the code counts) of the three forms of motivations were consistent or different between in-school and out-of-school contexts.

## Results

The theoretical components within the TCM, including motivation in-school and out-of-school contexts, the three social cognitive factors (attitude, subjective norm, PBC), and intention, emerged as higher-order themes in the thematic analysis. In general, most of the students understood sport injury prevention as doing warm-up, such as running laps and stretching. Few students mentioned cool down as a preventive measure. The details and English translations of quotations of the higher-order themes and their corresponding lower-order themes are presented in Table 2. Where quotations are provided, the participants’ reference is presented for gender (F = female and M = male) and group (G1-G17 = Group 1—Group 17).

**Table 2:** Themes and sub-themes.

Main theme	Sub-themes	Examples of Quotations	Code Count
Motivation			
1. Motivation in-school	Autonomous	“I do it (warm-up) to prevent injury” (M, G7) “Lower the chance of getting injury” (F, G11)	40
	Controlled	“Only do it when teacher ask us to do it” (F, G13) “Warm-up, absolutely will not be self- initiated” (M, G8)	83
	Amotivation	“I just do it (warm-up) but never questioned why” (F, G1)	6
2. Motivation out-of-school	Autonomous	“To relax the muscle” (F, G17) “(I do warm-up) because If you are injured, you cannot play in competition” (M, G4)	44
	Controlled	“The coach outside (school) will ask me to do it” (M, G7) “If my father is not around, I don’t need to do it” (F, G2)	20
	Amotivation	“When it comes to my mind/ attention I do it (warm-up)” (F, G2)	8
Social cognitive factors			
3. Attitude	Affective	Positive: “[interviewer: how do you feel about doing sport injury prevention?] It is quite okay… You feel comfortable after doing it” (F, G16) Negative: “(stretching) is painful, I don’t want to do it” (F, G17)	34
	Instrumental	Positive: “To improve performance” (M, G9) Negative: “It is the same whether you do it (warm-up) or not” (F, G9)	125
4. Subjective norms	Injunctive norms	Positive: “(people think that you are) very cool and professional to do stretching” (M, G14) Negative: “If you do it outside, people will look at you” (F, G5) No idea: “No one care about doing warm-up” (F, G1)	241
	Descriptive norms	Positive: “Nothing special, people next to me also do it (warm-up)” (F, G10) Negative: “You go out to play, people will not do it (warm-up) as well” (M, G7)	53
5. PBC	Positive	“if we have more time, we can do more; less time, we cannot not do it” (M, G12)	84
	Negative	“No confidence, if no one does it together, it is difficult” (F, G10)	53
6. Intention	Intention	“Yes, I injured my arm before, so I will need to stretch it” (M, G16)	45
	No intention	“No, why will I do it?” (F, G8)	41
7. Behaviors	In-school	“Jog for two laps” (M, G7) “Yes, we stretch every single time before doing sports (PE lessons)” (F, G2)	94
	Out-of-school	Positive: “I do a warm-up in swimming pool” (F, G1) Negative: “I start to play right away” (M, G7)	104

### Motivation

#### Motivation in-school

This theme refers to the motivation that students endorsed toward in-school sport injury prevention measures. The three main emergent subthemes were *autonomous motivation*, *controlled motivation*, and *amotivation*. Students reported being autonomously motivated to prevent in-school sport injuries when they self-endorsed the values or benefits of warm-up activities or exercises. They viewed warm-up exercises as preventing muscle pain, stiffness, sprain, sport injury or enhancing sport performance: “I want to protect myself” (M, G16), “(Why will you do sport injury prevention?) It is good to my body” (M, G11). Controlled motivation refers to the external demands, pressure, and pride satisfaction of doing sport injury prevention. Many students reported that they experienced controlled motivated to carry out the preventive measures in-school: “I do it (warm-up) only when teachers ask us to do it” (M, G9), “Sometimes it (warm-up) is compulsory, and so you need to do some to avoid being scolded (by teachers)” (F, G1). Sometimes, students did not know the reasons they engaged in sport injury prevention in-school. These quotes are under the themes of amotivation: “(What are the reasons that you do injury prevention in PE lesson?) No reason we just do it” (M, G17), “I do it (warm-up) because I have nothing else to do” (F, G4).

#### Motivation out-of-school

This theme specifically represents students’ motives to prevent sport injury in out-of-school context. A*utonomous motivation*, *controlled motivation*, and *amotivation* emerged as sub-themes. For the autonomous motivation, similar responses could be found in-school and out-of-school contexts. “I really want to do better in the competition” (F, G1), “(I want) to prevent cramping (in swimming)” (F, G5). Outside of school, students also attempted to prevent sport injury because of external reasons (controlled motivated): “Yes, I will do (preventive measures), I have training during summer, I do it when coach asks me to do it” (M, G13), “When my father is around I definitely need to do (a warm-up)” (F, G2). For amotivation, some of the students’ responses showed absence of motivation towards sport injury prevention out-of-school: “(So do you know why you do sport injury prevention?) I really don’t know” (M, G6).

### Social cognitive factors

#### Attitude

Attitude refers to the personal evaluation of sport injury prevention. This theme encompasses two sub-themes, *affective attitude* and *instrumental attitude*. Affective attitude represents whether the students enjoy performing the preventive measures. It is further subdivided into positive and negative affective attitude. Students used “Refreshing”, “Relaxing” (M, G6) and “Comfortable” (M, G16) to describe the positive feelings of warming-up. However, other students had different ideas: “(Doing a warm-up is) very boring” (M, G3), “That was very annoying is doing leg split” (F, G1).

Instrumental attitude refers to students’ assessment of the benefits of doing sport injury prevention. Many students did not consider preventive measures to be beneficial to them: “It is the same whether you do it (warm-up) or not” (M, G15), other terms like “Waste of time”, “Waste of energy” and “Useless” (M, G16) were also reported. In other cases, students believed injury prevention can “Reduce (muscle) pain”, “Reduce the chance of injury” and “Relax your muscle” (F, G5). A handful of students highlighted warm-up exercises can enhance their sport performance: “You will be more concentrated after warming-up”, “Improve competition performance” (M, G12).

#### Subjective norms

This theme refers to the perception of social appropriateness of sport injury prevention. *Injunctive norm* and *descriptive norm* emerged as lower-order themes. Injunctive norms referred to the perception of others’ approval or encouragement on preventing sport injury. Most students could not determine whether their significant others cared about their injury preventive behaviors (i.e., “No idea”): “My family members have no opinion (on whether I do warm-up)” (F, G2). Some felt that teachers, coaches and family members approved their behaviors: “If you do a lot (of warm-up exercises), people think that you are professional” (F, G9). Only a small number of students reported their social groups disapproved them to do sport injury prevention. They perceived others viewed them as “Pretending to be professional”, “Very weird” (M, G14), when they carried out the safety measures. Descriptive norms represented whether students’ significant others prevented sport injury or not. Both positive and negative descriptive norms were reported by the students: “Yes, they (parents) are the one to lead (the warm-up)” (F, G2), “Family members don’t do (warm-up exercises)” (M, G11).

#### PBC

This theme refers to students’ perceived ease or difficulty of adopting sport injury prevention. The two main emergent sub-themes were *positive PBC* and *negative PBC*. The majority of the students were confident in doing preventive measures: “It (doing warm-up exercises) is always easy” (M, G17). However, some students found it more difficult, “Very difficult, we need to do leg split” (F, G1). Environment was also reported to be a determinant of PBC, “It is easier to do if we have a mat”, “(It is easier to do), if we can turn on air conditioner” (F, G1). Students had negative PBC on injury prevention when the “Weather is hot”, “Not enough space” (M, G9).

#### Intention

Intention emerged as a higher order theme that refers to the students’ intention to engage in sport injury prevention. This theme was further divided into *intention* and *no intention*. Some students reported they are intended to participate in sport injury prevention: “Yes I will do some stretching after exercises” (M, G12), “I will do it in the training session in coming Thursday” (M, G5). For students who had no intention, they said “I will not do it” (F, G1), “No, why will I do it?” (F, G8).

### Behavior

Behavior was a higher-order theme that referred to the adoption of sport injury prevention in-school and out-of-school. All of the groups reported they needed to do warm-up exercises before PE class and a few students highlighted they do cool-down exercises. The warm-up in-school normally consisted of “standard stretching” (F, G2) and “Jogging for few laps” (M, G4). Besides doing warm-up exercises, PE teachers also taught “the correct techniques” (F, G6) and asked students to use safety equipment: “Knee pad” (M, G13) and “Shin guard” (M, G3). When students were out-of-school, approximately half of them said they would engage in sport injury prevention: “I do it (stretching) before swimming” (F, G5) and “Bring helmet and do warm-up before skating” (M, G6). The other half of the sample reported they would not do injury prevention out-of-school: “I jump right into to the swimming pool to swim” (M, G12), “I don’t think of putting on a helmet before cycling” (M, G4).

### Pattern of motivation between in-school and out-of-school

The code counts for in-school autonomous motivation, controlled motivation, and amotivation were respectively 40, 83, and 6; that for out-of-school were respectively 44, 20, and 8 respectively. A 2 x 3 chi-square test of independence (**χ**^**2**^ = 28.84, *p* < .01) indicated that patterns of motivation were different between the in-school and out-of-school contexts. Follow-up 2x2 chi-square tests indicated that controlled motivation was mentioned more often regarding in-school than out-of-school contexts (controlled and autonomous motivation x contexts: **χ**^**2**^ = 22.33, *p* < .01, odds ratio = 4.57; controlled motivation and amotivation x contexts: **χ**^**2**^ = 9.64, *p* < .01, odd ratio = 5.53). However, the frequency of autonomous motivation and amotivation were relatively consistent between the two contexts (autonomous motivation and amotivation x contexts: **χ**^**2**^ = .11, *p* = .74, odds ratio = 1.21).

## Discussion

The purpose of the current study was to explore junior secondary school students’ experience and perspectives of sport safety in-school and out-of-school context, with a particular focus on the meaning and content they applied to the psychological factors of sport injury prevention under the TCM [15–17]. The higher-order and lower-order themes emerged from thematic content analysis generally aligned with the motivational and social cognitive constructs of the model, but the pattern of motivation in-school and out-of-school context did not entirely support the proposition of the TCM as the patterns of controlled motivation did not appear to be consistent (or transferrable) between the two contexts. These results yet may provide information about the mechanisms underlying the process of trans-contextual transfer of motivation [12, 27–29].

### Motivation

The current data are supportive the presence of autonomous and controlled motivation, and amotivation for sport injury prevention among junior secondary school students [24, 27, 29]. However, when investigating the content of the quotes for autonomous motivation, we did not observe intrinsic motivation for sport injury prevention in either in-school or out-of-school contexts. This phenomenon may indicate that autonomously motivated students may participate in sport injury prevention because they think that it is useful or beneficial, rather than because it is fun. While “Having fun” has been regarded as an important factor that determines individuals’ adherence to sport injury prevention [14], and researchers also proposed that injury prevention programs should be more game-like [41] our current data suggest that students are not intrinsically motivated to participate in injury preventive measures. Although the absence of intrinsic motives for sport injury prevention is somewhat in line with the operationalization of autonomous motivation in the sport injury prevention version [24, 29] of treatment self-regulation questionnaire [42], our findings may raise further questions about the necessity, applicability, effectiveness, and practicality of promoting intrinsic motivation for sport injury prevention. Nevertheless, workshops and interventions can be provided to PE teachers and coaches, introducing ways to develop enjoyable sport injury prevention programme (e.g. jogging with a football, rotating leadership in leading dynamic stretching). Another effective strategy would be to enhance other autonomous forms of motivation, such as identified regulation. This would mean a focus on identifying the internally valued outcomes of injury prevention (e.g., being able to continue participating in exercise, avoiding lengthy rehab or visits to the physiotherapist), rather than promoting enjoyment of the exercises themselves.

Another noteworthy finding in this study concerns about content of amotivation for sport injury prevention. Amotivation, compared to autonomous and controlled motivation, was a theme that received less mention (expressed via codes), but its expressions in the quotations did not always appear to be maladaptive as it was described within SDT [19]. In this study, amotivated students were not aware of the reasons behind why they sported injury prevention, and they did not feel pressured to do so. However, follow-up questions about why indicated that (1) some students believed that it was easier to follow what it was told or what everyone else was doing, (2) or they just did it automatically or habitually when time allowed. The former case was more prevalent for in-school amotivation, and might reflect lack of true intention towards sport injury prevention, thus more vulnerable to dropout and low-awareness to sport injury prevention in some circumstances (e.g., unsupervised out-of-school conditions). It might also explain why the latter case (i.e., automaticity and habit) was more commonly found out-of-school amotivation. Such content related to amotivation might somewhat reflect concepts such as implicit attitude, implicit motivation, and habit, that growing amount of research have used them for the explanation of health behaviors [43–46]. Existing literature regarding the role of amotivation on sport injury prevention has been scarce, so it would be worthwhile for future studies to incorporate amotivation, and even other related factors (e.g., habit, implicit attitude) into the TCM [47].

The role of controlled motivation is another interesting observation. Our data indicated that students felt obliged to participate in safety measures, and felt that sport injury prevention was compulsory because they had to follow significant others’ (e.g., PE teachers in-school context, and coaches and parents in out-of-school context) instructions or comply with safety regulations. It seemed that students may not necessarily know the rationale behind performing sport injury prevention activities. Such a scenario is not ideal for behavioral adherence because in the absence of external demands or social pressure, individuals driven by controlled motivation are less likely than autonomous motivated individuals to adhere to sport injury prevention [19, 48], making them more vulnerable to behavioral dropout in out-of-school context. In the focus-group interview, there were several students who possessed in-school controlled motivation, but not out-of-school controlled motivation, and they also reported behavioral non-compliance in out-of-school context “I don’t do warm-up [outside school]”. This might be why the students were less likely to report controlled motivation for out-of-school injury prevention compared to in-school injury prevention.

Rates of autonomous motivation and amotivation (but not controlled motivation) for injury prevention were highly comparable between in-school and out-of-school contexts. These findings were in line with the tenets of TCM [15–17], it might provide implications for the trans-contextual transfer of motivation in the injury prevention context [23, 27, 28, 49]. Autonomous motivation and amotivation appeared to be more prevalent by participants than controlled motivation in the out-of-school context, so that might suggest that the transferability of autonomous motivation and amotivation is more effective than controlled motivation. Our data might, therefore, offer an explanation as to why some previous studies adopting the TCM reported non-significant [26] or relatively weaker association between controlled forms of motivation across contexts, as compared to that of autonomous forms of motivation [15, 50]. Yet, the answer has not been fully revealed as majority of the studies applying the TCM often use a composite score for motivation types from SDT (e.g., the relative autonomy index) rather than differentiated constructs [16, 51]. It might be important for future studies to examine the independent transferability of each type of motivation from SDT.

Our findings were consistent with previous studies examining the TCM in injury prevention regarding the transferability of autonomous motivation across contexts [23, 27]. According to SDT [18, 19] and prior studies in injury management [23, 27], autonomous motivation could be facilitated by satisfying individuals’ psychological needs of autonomy (feeling of choices and freedom), competence (feeling of being able to do what you want) and relatedness (feeling of being accepted, connected and cared for) [28, 52, 53]. However, questions remain on how PE teachers can provide the best support for satisfying students’ psychological needs in the injury prevention contexts, and answering this question require further analysis of PE teachers’ behaviors.

### Social cognitive factors

The current study provided evidence on students’ beliefs in sport injury prevention with themes consistent with the theoretical concepts of the social cognitive variables from the TCM, including attitude, subjective norms, and PBC [24, 29]. The sub-themes indicated there were positive and negative beliefs that governed students’ decision-making process for sport injury prevention. Our findings may be useful for understanding or even modifying the salient beliefs associated with students’ commitment to sport safety guidelines. Researchers and sport medicine practitioners should try to alter negative beliefs, such as affective (e.g., “painful feeling”) and instrumental (e.g., “waste of time”) attitudes, injunctive (e.g., “it makes me look weird in front of others”), descriptive norms (e.g., “None of my friends do it”), and PBC (e.g., “no time and space”) to try to draw students’ attention to the positive ones. For example, one common negative instrumental attitude is about the effectiveness of sport injury prevention. It refers to a misconception that sport injury is inevitable regardless of prevention, and previous studies have reported this belief was negatively related to self-determined (i.e., more autonomous, less controlled) motivation of injury prevention [27, 28]. Resolve this maladaptive belief by restating the evidence about the effectiveness of sport injury prevention on reducing the risk and severity of sport injuries [54]. A prior study in promoting helmet use among school-aged cyclists disseminated leaflets with persuasive messages constructed based on the TPB [20]successfully enhanced future helmet use by promoting change in the social cognitive variables [55]. Besides the three social cognitive variables, intention emerged as an independent theme in the present study, but the content regarding students’ future engagement in sport injury prevention rarely specified specific injury preventive behaviors, and when and how they would be performed. This finding might be due to the well-documented intention-behavior ‘gap’ in which intentions cannot fully predict behaviors because people do not act according to their intentions [56, 57]. Our data may imply that enriching the specification of intention that students formed for sport injury prevention might bridge the intention-behavior gap, and this could be done by fostering better action control, implementation planning, action/recovery self-efficacy [56–59]. Several behavioral change strategies have been proposed by the literature to tackle these variables, for example the “if, then” approach proposed by Chapman, Armitage [60]. Future studies could investigate the feasibility of applying these evidence-based behavioral change strategies in sport injury prevention contexts.

### Behaviors

Injury preventive behavior reported by junior secondary school students reported many strategies related to sport safety. However, pre-exercise warm-up and stretching dominated the content of this theme. Stretching during pre-exercise warm-up might not necessarily be the most appropriate method for sport injury prevention [61]. Some studies even suggested stretching could have negative effects on performance [62], and might have a non-significant impact on injury prevention [63]. Other types of preventive methods, such as neuromuscular training (e.g. FIFA 11+, iSPRINT) [54, 64, 65], eccentric strength training [66], resistance training [67] received increasing amount of evidence in supportive to their effectiveness on sport injury prevention. Our findings may imply that besides fostering better behavioral adherence, enhancing the knowledge of sport injury prevention among students and PE teachers (e.g., sport safety workshop, education seminar) might be critical to reducing the risk of sport injury, particularly in out-of-school unsupervised situations [10, 68].

### Limitations and future directions

A few limitations of the current study should be addressed to identify the boundaries of the study and stimulate further research. Our study adopted a qualitative approach focusing on the content of the psychological factors of TCM, and the frequency salient themes [69]. The cross-sectional nature of the study and qualitative data mean that we cannot draw causal inference on transfer of motivation, and the change in psychological variables within the TCM. A longitudinal study with cross-lagged panel design could examine the temporal relationship by testing the changes of TCM variables over time [70]. Another noteworthy limitation is related to the study sample. Although our study sample was recruited from only two local secondary schools in Hong Kong, the variation of participants’ personal backgrounds, school environment, sport culture, and region of residence could be restricted, so it might affect the generalizability of the findings to other populations. Future studies should replicate this line of work with diverse samples with participants from different backgrounds, and more importantly, in other behavioral contexts (e.g., physical activity, occupational injury prevention, rehabilitation, and education) where qualitative studies of the TCM have yet to be employed.

## Conclusion

The current study is the first qualitative study to explore junior secondary school students’ experience and perspectives on sport injury prevention, using TCM as a framework for investigation. Themes emerged from 17 focus group interviews were consonant with the constructs of the TCM, including in-school motivations, and out-of-school motivations, social cognitive factors, intention, and behavior regarding sport injury prevention. The frequency of codes for motivation could be explained by the tenets of the TCM’s regarding the transferability of motivation across contexts. The frequency of autonomous motivation and amotivation was highly consistent across the two contexts, but that of controlled motivation was significantly reduced in out-of-school context. The content of behavior also indicated the inadequacy of students’ knowledge of effective sport injury prevention techniques, and underscored the importance of sport safety education. Based on the findings of prior studies on the TCM in other behavioral contexts (e.g., occupational injury prevention), making goal-oriented safety objectives, promoting the pros of preventing sport injury, encouraging everyone to participate in injury prevention (including students’ family) and removing students’ barriers to do sport injury prevention (e.g. uneven surface, hot weather and time limit), might be possible solutions to enhance students’ adherence to engage in sport injury prevention [12, 28]. Future quantitative research is warrant to test the effectiveness of these strategies on students’ behavioral adherence towards sport injury prevention.

## Supporting information

S1 Appendix Interview schedule. (DOCX)
