# Supplementary material for: Sport injury prevention in-school and out-of-school? A qualitative investigation of the trans-contextual model
Source: PLoS One. 2019 Sep 6;14(9):e0222015. doi: 10.1371/journal.pone.0222015 (PMC6730904; doi:10.1371/journal.pone.0222015)
Supplement: S1 Appendix — (DOCX) [file pone.0222015.s001.docx]

**Appendix**

**Interview Schedule**

**Rapport Building and Sporting Experience**

1. Please share with us about your sport experience.

- What sport(s) do you do in-school and out-of-school context?
- Why do you do these sports (motivation)?
  - What drive you to do sport in-school and out-of-school contexts?
  - How do you feel when you do sport?
- Who are the people important to your sport participation?
  - How do they support or influence your sport participation?
  - How do you generally feel about them?

**Sport Injury Experience**

2. Do you know what is sport injury?

3. Have you experienced sport injury?

- How often?
- Please share with us the most severe sport injury experience you have had.
- Where did it happen? In-school or out-of-school contexts?
- Have you recovered? How?

4. What do you usually do for preventing sport injury? Why?

- In-school context
- Out-of-school context

**Psychological Need Support and Motivation**

5. In the PE lesson, what your PE teachers do for preventing sport injury? Why?

- Under his/her teaching, how do you generally feel about sport injury prevention in PE-class? Why?
- Do it for your own or guidelines/ school rule?
- Hard or easy?
- Not feeling strange or alone?

6. To you, what are the main reasons you would like to prevent sport injury in-school and out-of-school context?

- How much do you feel that it is something you WANT TO do? Why?
- How much do you feel that it is something you HAVE TO do? Why?
- How much do you feel that you DON’T KNOW WHY you do it? Why?

**Social Cognitive Factors**

7. What, if anything, do you believe are the advantages/ disadvantages of sport injury prevention?

8. Are there any individuals, or groups, who would approve/disapprove of your preventing sport injury?

- Approve/ Disapprove
- Are there any significant individuals or group would/ wouldn’t prevent sport injury?
- What do you, or other athletes feel when you do or do not prevent sport injury?

9. What factors or circumstances would make it easy/difficult/ impossible for you to consider sport injury prevention?

- Easy/ difficult/ impossible

10. In consideration of all the positive and negative factors of sport injury prevention, how much do you want to do it in the foreseeable future, e.g., next month?

- Willingness/ planning/ effort

This concludes the focus group questions. Do you have any questions or further information to add?

Thank you for your time
